# Infantile Mortality

**Published:** 1898-09

**Authors:** 


### Infantile Mortality.

Dr. Letters has contributed an article to the *Dublin Journal of Medical Science* upon the mortality of infants in Ireland, in which he says that they are allowed to perish in myriads by feeding-bottles with foul rubber tube-fittings, by farinaceous substances which ferment and decompose as soon as ingested, by neglect, by exposure, by deprivation of the natural nutriment, by preventable causes like premature birth, by preventable diseases like diarrheas and diseases of the respiratory organs. Infants want protection against that killing ignorance which does them to death by artificially induced diarrheas, by convulsions from improper feeding, by marasmus from starvation. Let the question of the saving of infant life receive from the profession that measure of study to which it is justly entitled, let the best means be formulated to stem the infantile death current in our populous urban centers, and let the legislature be urged to deal with the subject in a spirit of humane comprehensiveness. It is unfortunately true that Ireland is not the only country to which these words might be applied.

